# Retrotransposon Silencing by piRNAs: Ping-Pong Players Mark Their Sub-Cellular Boundaries

**DOI:** 10.1371/journal.pgen.1000770

**Published:** 2009-12-11

**Authors:** Shinichiro Chuma, Ramesh S. Pillai

**Affiliations:** 1 Institute for Frontier Medical Sciences, Kyoto University, Kyoto, Japan; 2 European Molecular Biology Laboratory, Grenoble, France; Stanford University School of Medicine, United States of America

Germ cells of many animals exhibit characteristic cytoplasmic structures—termed germ granules or nuage—which are ribonucleoprotein (RNP) amorphous aggregates without limiting membranes and are often closely associated with nuclei or mitochondria [1]. In several model animals, such as *Drosophila*, *Caenorhabditis elegans*, and *Xenopus*, studies on germ granules have mainly focused on their asymmetric partitioning to prospective germ cells in early embryogenesis, leading to a supposition that the RNP structures contribute to the establishment of the germline. In mammals, in contrast, germ granules become discernible at later stages of germ cell differentiation, i.e., in spermatogenesis and oogenesis, and are not asymmetrically segregated. Thus, their possible function seems different from those in early embryos of other species. Despite the difference, germ granules of diverse animals, including mammals, not only share morphological similarities, but their molecular compositions are also conserved [2], suggesting a common and essential function in the germline, which however remained unanswered for many years.

Recently, almost a century after the first description of germ granules [1], this longstanding enigma, or at least a part of it, is finally being unraveled. Accumulating evidence now points to a close association of germ granules with retrotransposon control and, especially, the piwi-small RNA pathway. Germ cells of many animals, from worms to mammals, are equipped with specific members of the argonaute subfamily, the piwi proteins, which associate with piwi-interacting small RNAs (piRNAs), and this small RNA pathway is critical for retrotransposon silencing in the germline (and gonadal soma in *Drosophila*) [3]. In mice, *Mili/Piwil2* and *Miwi2/Piwil4*, two mouse *piwi* members, are central to the feed-forward or ping-pong production of piRNAs from retrotransposon and other cellular transcripts in the male germline, and their loss-of-function mutations lead to deregulation of *Line-1* and *IAP* retrotransposons, resulting in male sterility with meiotic spermatocyte defects [4]–[6].

In this issue of *PLoS Genetics*, Aravin and colleagues in the Bortvin and Hannon groups report the remarkable finding that both piwi proteins exhibit distinct subcellular compartmentalization in fetal prospermatogonia/gonocytes, wherein retrotransposon silencing is established during male germline development [7]. MILI localizes to inter-mitochondrial cement (also called inter-mitochondrial material/bar/cloud etc.), a form of germ granules commonly observed in divergent animals, and MIWI2 accumulates at processing bodies (P-bodies), an mRNP assembly widely conserved in eukaryotes from yeast to humans and whose presumed function is general RNA degradation/translational control, including those mediated by miRNAs and siRNAs [8]. These distinct MILI and MIWI2 granules contain tudor domain containing proteins TDRD1 and TDRD9, respectively, which also operate in the piwi pathway to suppress retrotransposons [9]–[12]. The discrete localization of MILI-TDRD1 to germ granules and MIWI2-TDRD9 to processing bodies indicates that the two RNP complexes, which are often found in close proximity, represent functionally separate assemblies of the small RNA machineries that likely co-operate and interdependently function in piRNA biogenesis and retrotransposon silencing.

Aravin et al. add another key player, Maelstrom (MAEL), in the piwi-small RNA pathway. MAEL has a HMG box and a domain homologous to DnaQ-H 3′-5′ exonuclease, and is conserved from protists to mammals [13]–[16]. The authors show that the MAEL protein in mice is specifically colocalized with MIWI2-TDRD9 granules in the cytoplasm of prospermatogonia, in addition to their accumulation in the nucleus. The MIWI2-TDRD9-MAEL complex was shown to correspond to a subpopulation of processing bodies as identified by P-body markers DCP1a, DDX6, XRN1, and GW182. They named this subpopulation of processing bodies containing MIWI2-TDRD9-MAEL “piP-bodies” and inter-mitochondrial cement localized with MILI-TDRD1 “pi-bodies”. The latter term was recently also proposed for *Drosophila* nuage enriched with piwi pathway components, retroelement transcripts, and processing body components [17]. In mice, the MAEL localization is dependent on the *Mili* function, but not vice versa, similarly to the requirement of *Mili* for MIWI2 and TDRD9 localizations [10],[18], and then *Mael* regulates the assembly of MIWI2 and TDRD9 onto piP-bodies. Thus, MAEL acts downstream of MILI and upstream of MIWI2-TDRD9 with respect to the subcellular compartmentalization in fetal prospermatogonia in mice.

Previously, the authors reported that *Mael* gene–targeted mice are male-sterile and show a strong activation of *Line-1* retrotransposon in postnatal testes [16]. Now, they extend their analysis of *Mael* mutants to fetal prospermatogonia and uncover a striking finding that piRNA production is severely impaired at embryonic day 16.5 (E16.5) with transposon-derived piRNAs being virtually absent in *Mael* mutants, but the defect largely recovers at postnatal day 2 (P2), while secondary piRNAs, which preferentially load onto MIWI2, are under-represented by several-fold. Together with the precise colocalization of MAEL with MIWI2 at piP-bodies and their epistatic relationship, the authors argue that the *Mael* mutation affects the MIWI2 pathway in the ping-pong production of piRNAs, resulting in a delayed accumulation of piRNAs with decreased secondary piRNA signatures. In *Mili*, *Miwi2*, and *Tdrd1* mutants, the biogenesis and/or sequence profile of piRNAs are also significantly impacted, and de novo DNA methylation of retrotransposon loci, which usually takes place in fetal prospermatogonia in the male germline, is severely impaired [4]–[6],[10],[12],[19]. However, in *Mael* mutants, DNA methylation at *Line-1* retrotransposon loci examined is only moderately decreased in prospermatogonia at E16.5, which recovers by E18.5 and then the difference is not detectable at neonatal P2. The authors correlate this unexpected lag and recovery of de novo DNA methylation with delayed nuclear accumulation of MIWI2 in *Mael* mutant prospermatogonia and suggest that MAEL “facilitates” MIWI2-dependent steps of the piRNA pathway. These observations provide unprecedented insights that the coordinated and ordered operations of the piwi pathway components at around embryonic day E16.5—when or just after fetal prospermatogonia become arrested at the G1/G0 stage and DNA methylation reprogramming commences—are essential in the establishment of retrotransposon silencing in the male germline in mice. In these processes, *Maelstrom* is a critical modulator that acts in the MIWI2 pathway. Meanwhile, one key question that arises from this study is, given the recovery of DNA methylation in the *Mael* mutant, what then triggers the later retrotransposon activation in postnatal spermatogenesis as was reported previously [16]? One possibility might be that histone modifications are affected independent of DNA methylation at the locus examined. Alternatively, *Mael* might have an additional function separate from other piwi pathway components so far identified. A recent study in *Drosophila* actually showed that *Mael* regulates Bag-of-marbles via repression of miR-7 and ensures proper differentiation of spermatocytes [20]. It remains to be addressed whether this novel function of *Mael* in the miRNA pathway is retained across species, in addition to its conserved role in the piwi machinery.

The study by Aravin et al. reveals that germ granules, namely pi-bodies, and a germline analogue of processing bodies, piP-bodies, are cytoplasmic compartments where piwi pathway components assemble. The next questions are how and why these components are differently sorted-out into distinct subcellular domains, and what is the underlying molecular mechanism wherein the two RNP complexes cooperate in the piRNA biogenesis, which is intimately linked to retrotransposon silencing at both transcriptional and post-transcriptional levels. It is important to note that current experiments performed with fixed tissue sections give us a static image of potentially dynamic interactions between the two RNP complexes. Development of suitable cell culture systems that recapitulate the piRNA pathway and the use of live-cell imaging techniques will help explore this further. It is also currently unclear whether these RNP assemblies are functional prerequisites for the piwi-small RNA pathway operation, or such cytoplasmic aggregations are consequences and by-products of normal cellular metabolism. Indeed, in somatic cells, microscopically visible processing bodies are not required for proper functioning of the small RNA pathway [21]. Another evident but untouched issue is that “pi-bodies” in prospermatogonia correspond to “inter-mitochondrial cement” located in the midst of mitochondrial clusters (Figure 1). At present, we do not have any experimental clues to discuss whether there would possibly be any correlation between the piwi pathway and mitochondria. The physiological function(s) of germ granules is one of the classic but still enigmatic problems in developmental and cell biology and remains to be fully determined. Further characterization of germline RNPs and the piwi-small RNA pathway associated there would uncover an intriguing molecular mechanism(s) that is present but still hidden within the germline.

**Figure 1:**
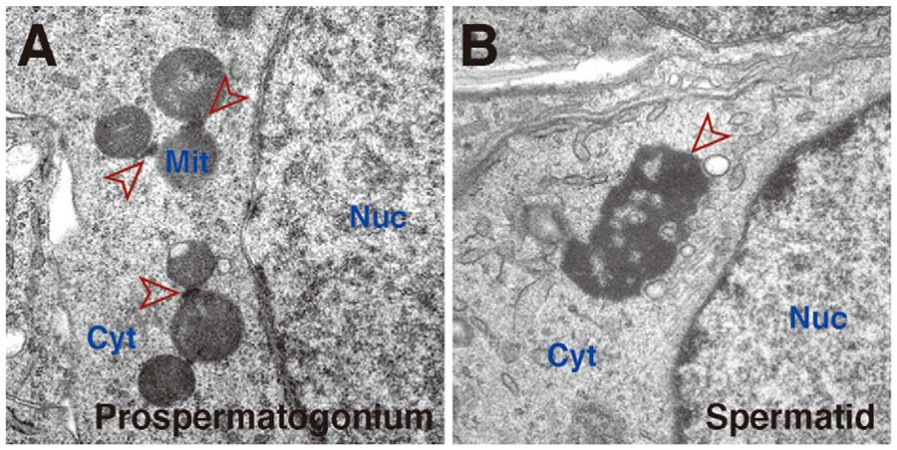
Germinal granules/nuage in mouse germ cells. Electron microscopy of a fetal prospermatogonium (A) and postnatal spermatid (B). In (A), inter-mitochondrial cement structures are seen as fine electron-dense material among mitochondria (arrowheads). Inter-mitochondrial cement is also seen in postnatal spermatogonia, spermatocytes and in growing oocytes. In (B), a chromatoid body (arrowhead), a specialized form of germinal granules/nuage, is seen in the cytoplasm independently of mitochondria. Chromatoid bodies are much larger in size than inter-mitochondrial cement and are observed mostly as one or two solitary aggregates in haploid spermatids. Both inter-mitochondrial cement and chromatoid bodies contain piwi-pathway components. Nuc, nucleus; Cyt, cytoplasm; Mit, mitochondria.

## References

[1] Eddy EM (1975). Germ plasm and the differentiation of the germ cell line.. Int Rev Cytol.

[2] Chuma S, Hosokawa M, Tanaka T, Nakatsuji N (2009). Ultrastructural characterization of spermatogenesis and its evolutionary conservation in the germline: germinal granules in mammals.. Mol Cell Endocrinol.

[3] Girard A, Hannon GJ (2008). Conserved themes in small-RNA-mediated transposon control.. Trends Cell Biol.

[4] Aravin AA, Sachidanandam R, Girard A, Fejes-Toth K, Hannon GJ (2007). Developmentally regulated piRNA clusters implicate MILI in transposon control.. Science.

[5] Carmell MA, Girard A, van de Kant HJ, Bourc'his D, Bestor TH (2007). MIWI2 is essential for spermatogenesis and repression of transposons in the mouse male germline.. Dev Cell.

[6] Kuramochi-Miyagawa S, Watanabe T, Gotoh K, Totoki Y, Toyoda A (2008). DNA methylation of retrotransposon genes is regulated by Piwi family members MILI and MIWI2 in murine fetal testes.. Genes Dev.

[7] Aravin AA, van der Heijden GW, Casteneda J, Vagin VV, Hannon GJ Cytoplasmic compartmentalization of the fetal piRNA pathway in mice.. PLoS Genet.

[8] Eulalio A, Behm-Ansmant I, Izaurralde E (2007). P bodies: at the crossroads of post-transcriptional pathways.. Nat Rev Mol Cell Biol.

[9] Kojima K, Kuramochi-Miyagawa S, Chuma S, Tanaka T, Nakatsuji N (2009). Associations between PIWI proteins and TDRD1/MTR-1 are critical for integrated subcellular localization in murine male germ cells.. Genes Cells.

[10] Vagin VV, Wohlschlegel J, Qu J, Jonsson Z, Huang X (2009). Proteomic analysis of murine Piwi proteins reveals a role for arginine methylation in specifying interaction with Tudor family members.. Genes Dev.

[11] Wang J, Saxe JP, Tanaka T, Chuma S, Lin H (2009). Mili interacts with tudor domain-containing protein 1 in regulating spermatogenesis.. Curr Biol.

[12] Reuter M, Chuma S, Tanaka T, Franz T, Stark A (2009). Loss of the Mili-interacting Tudor domain-containing protein-1 activates transposons and alters the Mili-associated small RNA profile.. Nat Struct Mol Biol.

[13] Clegg NJ, Frost DM, Larkin MK, Subrahmanyan L, Bryant Z (1997). Maelstrom is required for an early step in the establishment of Drosophila oocyte polarity: posterior localization of grk mRNA.. Development.

[14] Costa Y, Speed RM, Gautier P, Semple CA, Maratou K (2006). Mouse MAELSTROM: the link between meiotic silencing of unsynapsed chromatin and microRNA pathway?. Hum Mol Genet.

[15] Zhang D, Xiong H, Shan J, Xia X, Trudeau VL (2008). Functional insight into Maelstrom in the germline piRNA pathway: a unique domain homologous to the DnaQ-H 3′-5′ exonuclease, its lineage-specific expansion/loss and evolutionarily active site switch.. Biol Direct.

[16] Soper SF, van der Heijden GW, Hardiman TC, Goodheart M, Martin SL (2008). Mouse maelstrom, a component of nuage, is essential for spermatogenesis and transposon repression in meiosis.. Dev Cell.

[17] Lim AK, Tao L, Kai T (2009). piRNAs mediate posttranscriptional retroelement silencing and localization to pi-bodies in the Drosophila germline.. J Cell Biol.

[18] Aravin AA, Sachidanandam R, Bourc'his D, Schaefer C, Pezic D (2008). A piRNA pathway primed by individual transposons is linked to de novo DNA methylation in mice.. Mol Cell.

[19] Chuma S, Hosokawa M, Kitamura K, Kasai S, Fujioka M (2006). Tdrd1/Mtr-1, a tudor-related gene, is essential for male germ-cell differentiation and nuage/germinal granule formation in mice.. Proc Natl Acad Sci U S A.

[20] Pek JW, Lim AK, Kai T (2009). Drosophila maelstrom ensures proper germline stem cell lineage differentiation by repressing microRNA-7.. Dev Cell.

[21] Filipowicz W, Bhattacharyya SN, Sonenberg N (2008). Mechanisms of post-transcriptional regulation by microRNAs: are the answers in sight?. Nat Rev Genet.

